# The Immunoglobulin Domain of SISS-1/EGF is Required for its Function

**DOI:** 10.17912/micropub.biology.001842

**Published:** 2025-09-14

**Authors:** Jesse G Jones, Cheryl Van Buskirk

**Affiliations:** 1 Biology, California State University, Northridge, Northridge, California, United States

## Abstract

Epidermal growth factor (EGF) signaling plays roles in development and physiology across the animal kingdom. In the nematode *C. elegans* , a single EGF receptor (EGFR) and two EGF family ligands have been characterized. LIN-3 /EGF is required for a variety of developmental processes as well as ovulation, and SISS-1 /EGF promotes a damage-responsive quiescent state known as stress-induced sleep. Like all EGF family ligands, SISS-1 and LIN-3 are produced as transmembrane proteins with an extracellular EGF-like domain, known for its function in receptor binding. The ectodomain of SISS-1 , but not of LIN-3 , also contains an Immunoglobulin-like (Ig) domain, putting it into a class of Ig-EGFs that includes *Drosophila* Vein and certain vertebrate Neuregulins. The function of the Ig domain within Ig-EGFs appears to vary. Here, we investigate the SISS-1 Ig domain and show that it is essential for stress-induced sleep.

**Figure 1. siss-1(ΔIg) animals are severely defective in stress-induced sleep:**
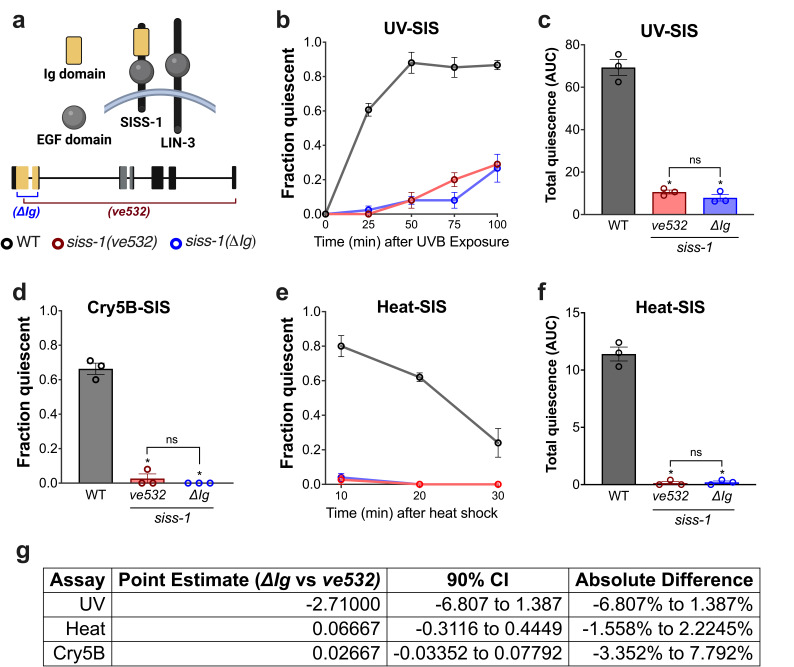
(a) Schematic of SISS-1 and LIN-3 topology and domains (top) and schematic of *siss-1* ( *ΔIg)* and *siss-1 ( ve532 )* deletion alleles (bottom). Both LIN-3 and SISS-1 are predicted membrane-bound proteins containing an extracellular EGF domain; SISS-1 also has an Ig domain (The Uniprot Consortium 2025). The cell membrane is depicted as an arch. (b-f) Assays of stress-induced sleep: (b) Fraction of animals quiescent following 50s of UV-B irradiation. (c) Area under the curves (AUC, total quiescence) for panel b. *P<0.01 vs wild-type N2 (WT), ns P=0.4698, Dunnett's T3 multiple comparisons test. (d) Fraction of animals quiescent at a single time point 10 min after a 10-min Cry5B exposure. *P<0.0001, ns P=0.4966, two-sided Fisher's exact test. (e) Fraction of animals quiescent following a 20-min 35°C heat exposure. (f) Area under the curve calculations for panel e. *P=0.0064, ns P=0.9714, Dunnett's T3 multiple comparisons test. Each data point in panels c, d, and f represents one trial of at least 22 animals. Error bars represent SEM. (g) Absolute percent difference of *ΔIg* vs *ve532* alleles, calculated from 90% CIs generated from an unpaired t test with Welch's correction (UV-SIS and Heat-SIS AUC) or the Newcombe-Wilson method with continuity correction (Cry5B-SIS). Point estimates were calculated as either a difference ( *ΔIg* vs *ve532* ) in AUC mean (UV and Heat) or a difference in quiescence proportions (Cry5B). Cry5B-SIS proportions were calculated by pooling data from all three trials, providing a single proportion for each genotype.

## Description

Members of the epidermal growth factor (EGF) family of ligands activate receptor tyrosine kinases of the ErbB/EGF receptor (EGFR) family and are found across Bilateria (Stein & Staros 2006). For decades, the only known *C. elegans* EGF was LIN-3 (Hill & Sternberg 1992), which is required for a variety of developmental events (Hill & Sternberg 1992; Chamberlin & Sternberg 1994; Chang et al. 1998) as well as for ovulation (Clandinin et al. 1999). Recently, our group identified a second *C. elegans* EGF, SISS-1 (Hill et al. 2024), that is required for a damage-responsive sleep state known as stress-induced sleep (SIS). While SISS-1 and LIN-3 appear to have non-overlapping roles, overexpression of either EGF can promote sleep via activation of LET-23 /EGFR within sleep-promoting neurons (Van Buskirk & Sternberg 2007; Konietzka et al. 2020; Hill et al. 2024).

EGF family ligands are produced as transmembrane proteins that undergo processing to release a soluble EGF-like domain (EGF domain), which binds to and activates EGF receptors (Singh et al. 2016). A subset of EGF ligands including *C. elegans* SISS-1 (Fig. 1a), *Drosophila* Vein, and some vertebrate Neuregulins also contain a membrane-distal immunoglobulin-like domain (IgD) (The UniProt Consortium 2025), the function of which is less understood. The IgD of mammalian Neuregulin 1 appears to potentiate EGFR activation by concentrating the ligand within the extracellular matrix (Li & Loeb 2001), while the IgD of Vein has little impact on its endogenous function but appears to mitigate the toxicity of ectopically expressed Vein (Donaldson et al. 2004), potentially by suppressing inappropriate receptor activation. Thus, different IgDs appear to modify EGF signaling in different ways. Here we investigate the role of the SISS-1 IgD.

To test the function of the SISS-1 immunoglobulin domain, we examined a CRISPR-generated IgD deletion allele, *siss-1 ( syb7881 )* a.k.a. *siss-1 (ΔIg)* (Fig. 1a), for its impact on stress-induced sleep (SIS), comparing it to wild type and to the null allele *siss-1 ( ve532 )* (Fig. 1a; Hill et al. 2024). We assayed the sleep response to three different stressors: UV-B radiation (Fig. 1b,c), Cry5B pore-forming toxin (Fig. 1d), and noxious heat (Fig. 1e,f). In each case, we found *siss-1 (ΔIg)* animals to be severely SIS-defective, and not significantly different from the *siss-1* null mutant. To explore the similarity of the *siss-1* mutant phenotypes, we generated 90% confidence intervals (CIs) and converted them to absolute percent differences, approximating the true differences in SIS responses between the *ΔIg* and *ve532* populations. These differences appear to be very small (Fig. 1g), but whether they are biologically negligible cannot be resolved here.

Taken together, our data indicate that the Ig domain is essential for SISS-1 function, contrasting with previously characterized Ig-EGFs. Mechanistically, the SISS-1 IgD may be required for protein folding, trafficking, processing, stability, or EGFR interaction. It is difficult to say why SISS-1 /EGF might have evolved this IgD dependence, but we speculate that it may represent a layer of regulation of SISS-1 activity in addition to its known stress-responsive shedding (Hill et al. 2024). As SISS-1 and its sheddase ADM-4 are widely expressed (Taylor et al. 2021; Ho et al. 2022), the potentially widespread availability of SISS-1 may necessitate another regulatory step. For example, the Ig domain might, like ADM-4 , be stress-responsive and required for SISS-1 signaling, ensuring that SIS is triggered only by critical tissue damage.

## Methods

**Growth conditions:** Strains were grown on nematode growth media (NGM) plates seeded with a thin lawn of OP50 *E. coli* ( OP50 NGM plates) and kept anywhere between 16-23°C. For heat-SIS experiments, strains were grown at room temperature (23°C) to avoid exposing animals to temperature changes during handling prior to heat shock.

**Standard for scoring stress-induced sleep:** Following exposure of at least 30 fed young adult animals to stressor, plate lids were removed for the remainder of the assay. To score SIS, plates were gently moved to the center of the microscope stage and left undisturbed for 30-60 seconds prior to scoring. Animals were then viewed at 250x magnification and a minimum of 22 were scored for sleep. To minimize bias, the first 22-30 clearly visible animals with mouth on food were selected for scoring. Animals showing complete cessation of movement and pharyngeal pumping over a 3-second observation were scored as quiescent. Animals showing any movement and/or pharyngeal pumping were scored as awake. The experimenter was blind to genotype for all assays.

**Cry5B-SIS:** For Cry5B exposure, animals were transferred to plates containing 1 mM IPTG and 60 µg/ml carbenicillin that had been seeded at least one month prior with JM103 *E. coli* carrying an IPTG-inducible Cry5B transgene (Marroquin et al. 2000). After 10 min of Cry5B exposure, animals were transferred back to OP50 NGM plates, given 10 min of recovery time, and scored at a single time point for SIS.

**UV-SIS:** Animals were transferred onto 60x15 mm OP50 NGM plates (15 mL media), and plates were placed lid-side down on a UVP M-10E (50 mW/cm ^2^ ) transilluminator. Animals were exposed to 302 nm (UV-B) for 50 sec. To avoid potential variation in lid thickness, the same lid was used for all UV treatments.

**Heat-SIS:** Animals were transferred to 35 mm diameter OP50 NGM plates (5 mL media). Plates were sealed with parafilm and placed agar-side down in a circulating water bath (35°C) for 20 min. To end the heat shock, plates were placed on frozen LabArmor beads for 1 min, which brings the agar to room temperature.

**Generation of *siss-1 ( syb7881 )* :** Uniprot.org annotation (release 2025_03) identifies the SISS-1 immunoglobulin domain as extending from Pro11 to Arg109 (The UniProt Consortium 2025). CRISPR-Cas9 (SUNYbiotech, Precise Sequence Deletion service) was used to generate a deletion of the corresponding nucleotides 46-390 of the *siss-1 b* transcript. The EGF domain of SISS-1 is annotated to start at Asp119 (The UniProt Consortium 2025).

**Statistics and Data:** Graphing, figure panel arrangement, and statistical analyses were performed using GraphPad Prism version 10.3.1 for macOS, GraphPad Software, Boston, Massachusetts USA, www.graphpad.com .

## Reagents

**Table:** 

Strain	Genotype	Source
N2	wild isolate	CGC
RG3032	*siss-1 ( ve532 )* [LoxP + *myo-2* p::GFP:: unc-54 3' UTR + *rps-27* p::neoR:: unc-54 3' UTR + LoxP]) IV	CGC
PHX7881	*siss-1 ( syb7881 )* IV. CRISPR deletion of nucleotides 46-390 of the *siss-1 b* transcript	CVB

CGC = Caenorhabditis Genetics Center, CVB = Van Buskirk lab.
